# Importancia del compromiso del personal sanitario para la satisfacción de los pacientes en Atención Primaria

**DOI:** 10.1016/j.aprim.2022.102281

**Published:** 2022-02-07

**Authors:** María Carla Del Estal García, Santiago Melián González

**Affiliations:** a Universidad de Las Palmas de Gran Canaria, Las Palmas de Gran Canaria, España; b Grupo de Investigación en Emprendimiento, Empresa Digital e Innovación. Universidad de Las Palmas de Gran Canaria, Las Palmas de Gran Canaria, España

**Keywords:** Satisfacción del paciente, Compromiso organizativo, Calidad asistencial, Atención primaria, Gestión de personal, Patient satisfaction, Organizational commitment, Quality of care, Primary care, Personnel management

## Abstract

Conocer en qué medida el compromiso organizativo de los profesionales sanitarios de Atención Primaria es importante para la satisfacción de los pacientes. Estudio observacional, de corte transversal, cuantitativo, retrospectivo, y no controlado. Cuarenta centros de Atención Primaria pertenecientes al área de salud de Gran Canaria. Atención primaria. Seiscientos diecisiete profesionales sanitarios (46% médicos y 54% enfermeros) y 1.537 usuarios de los centros de Atención Primaria (35,39% hombres y 64,60% mujeres). Muestreo no probabilístico por conveniencia. Para evaluar el compromiso organizativo se usó un cuestionario autoadministrado a profesionales sanitarios de centros de Atención Primaria. La satisfacción de los pacientes se obtuvo mediante encuesta telefónica y las variables de control a partir de fuentes secundarias. Los datos se analizaron mediante regresión lineal múltiple. Para profundizar en los resultados se usaron también entrevistas semiestructuradas. El entorno socioeconómico de los centros de salud no influye en la satisfacción de los pacientes. El compromiso organizativo de los trabajadores influye positivamente en la satisfacción de los usuarios de los centros de salud. También se encontró que los profesionales más comprometidos tienen menos disponibilidad en sus agendas para citas a corto plazo. Las entrevistas sugieren que se debe a que dedican más tiempo a sus pacientes, lo cual hace que estén más satisfechos. El compromiso organizativo afectivo de los profesionales sanitarios es una actitud que permite mejorar la satisfacción de los pacientes, por ello, los gestores sanitarios deberían emplear prácticas dirigidas a fortalecer dicha actitud.

## Abstract (en)

To know to what extent the organizational commitment of primary care health professionals is important for patient satisfaction. Observational, cross-sectional, quantitative, retrospective, and uncontrolled study. 40 primary care centers belonging to the health area of Gran Canaria. Primary care. 617 health professionals (46% doctors and 54% nurses) and 1,537 users of primary care centers (35.39% men and 64.60% women). Non-probability sampling for convenience. To evaluate organizational commitment, a self-administered questionnaire was used for health professionals in primary care centers. Patient satisfaction was obtained by telephone survey and control variables from secondary sources. The data were analyzed by multiple linear regression. To deepen the results, semi-structured interviews were also used. The socioeconomic environment of the health centers does not influence patient satisfaction. The organizational commitment of the workers has a positive influence on the satisfaction of the users of the health centers. It was also found that the most committed professionals have less availability in their schedules for short-term appointments. The interviews suggest that it is because they spend more time with their patients, which makes them more satisfied. The affective organizational commitment of health professionals is an attitude that allows improving patient satisfaction, therefore, health managers should use practices aimed at strengthening this attitude.

## Introducción

En muchos países, la atención primaria es la puerta de entrada de los usuarios al sistema sanitario, considerándose parte esencial o nuclear del mismo^1^. Los países con una atención primaria de calidad presentan mejores índices de salud, mayor equidad en el reparto de recursos y muestran un sistema sanitario más eficiente, estimándose que alrededor del 80% de las patologías son resueltas en dicho nivel asistencial^2^.

Como consecuencia de lo anterior, las administraciones sanitarias aspiran a que los centros de salud alcancen distintos objetivos relacionados con una atención primaria eficaz, eficiente e integral^1^, ^3^. Para ello, es importante considerar la experiencia y las percepciones de los usuarios de los centros, con el fin de lograr una población satisfecha^4^, ^5^, ^6^. Tal y como se ha demostrado, la insatisfacción de los pacientes se relaciona con un aumento del coste de la atención sanitaria, la ineficacia de los tratamientos y con un número excesivo de peticiones de pruebas complementarias^7^, ^8^. En cambio, un paciente satisfecho buscará ayuda sanitaria de manera activa cuando sea necesario, cumplirá en mayor medida con las recomendaciones médicas y, a la hora de tomar decisiones con respecto a su tratamiento, mostrará un comportamiento más condescendiente^7^. Por ello, en los últimos años, la satisfacción de los pacientes ha recibido un gran reconocimiento como medida de calidad en el sector público^6^, considerándose un resultado de suma importancia para los servicios de salud^6^, ^7^, ^9^.

En el caso de los clientes de una empresa, su satisfacción se define como la evaluación que los mismos realizan sobre si el producto o servicio ha cumplido con sus necesidades o expectativas^10^. En el ámbito sanitario la satisfacción de los pacientes se ha conceptualizado de manera similar^9^. Según muestra la evidencia, la dimensión más importante de la satisfacción de los pacientes es la referida al comportamiento de los médicos y enfermeros. Se encuentra por encima de la satisfacción con los siguientes aspectos de los servicios sanitarios: los resultados clínicos, la accesibilidad, la demora en las salas espera, y sobre en qué medida los servicios coinciden con las necesidades de los pacientes^9^, ^11^. Por ejemplo, variables como la amabilidad, la simpatía y el apoyo emocional de los profesionales sanitarios son más valoradas que la propia atención técnica^12^, ^13^.

De cara a conocer qué determina la satisfacción de los pacientes, todo lo anterior sugiere estudiar la actitud de los profesionales sanitarios y, en particular, su compromiso organizativo. El motivo es la evidencia disponible, a partir de estudios realizados en entornos no sanitarios, sobre la influencia positiva del compromiso de los trabajadores en la satisfacción del cliente^14^, ^15^, ^16^. A pesar de esto último, esta relación no ha sido estudiada en profundidad en el ámbito sanitario.

El compromiso organizativo describe la fuerza de la identificación y el apego de una persona con una organización. Existe consenso en cuanto a su multidimensionalidad. Concretamente, se compone de tres dimensiones. El compromiso normativo, que refleja la obligación que siente el trabajador hacia la organización. El compromiso de continuidad, referido a la percepción de los trabajadores de los costes asociados a dejar la organización. Y, por último, el compromiso afectivo, que hace referencia al apego emocional, a la identificación y a la implicación del empleado con la organización. Esta última dimensión es la que ha mostrado estar directamente relacionada con la satisfacción del cliente^17^, ^18^. A su vez, la relación entre compromiso organizativo afectivo y rendimiento en el trabajo está constatada, así como con las variables comportamiento cívico en el puesto de trabajo, asistencia, y bajos absentismo, estrés y conflicto en la conciliación trabajo-familia, respectivamente^19^, ^20^. Por ello, en el caso de puestos de trabajo asistenciales, es esperable que el compromiso afectivo de los trabajadores influya en la satisfacción de los que reciben el servicio.

Por tanto, el objetivo de esta investigación es determinar, en el ámbito de la atención primaria, en qué medida el compromiso organizativo afectivo del personal sanitario de los centros de salud es relevante para la satisfacción de los pacientes.

## Metodología

### Diseño del estudio

Estudio observacional, de corte transversal, cuantitativo, retrospectivo y no controlado.

### Contexto

El estudio se realizó en 2019 en el Área de Salud de la isla de Gran Canaria, España. El Área de Salud consta de 40 centros de salud de atención primaria que dan asistencia a una población de 806.783 personas.

### Participantes

Personal sanitario y usuarios pertenecientes a los centros de salud del Área de Salud de Gran Canaria. El personal sanitario incluía médicos y enfermeros que llevaran como mínimo un año de antigüedad en el centro de salud.

### Variables y fuentes de datos

Los datos referentes a la satisfacción de los pacientes corresponden a 2019 y fueron cedidos por la Consejería de Sanidad de la Administración Autonómica del Gobierno de Canarias. La satisfacción de los pacientes se obtuvo a través de entrevistas telefónicas realizadas por el personal técnico dicha Consejería. La satisfacción se evaluó mediante una pregunta sobre satisfacción global. Esta se contestaba mediante un único ítem de escala tipo Likert de diez puntos, en la que 1 era totalmente insatisfactorio y 10 totalmente satisfactorio. Aparte, como variable objetiva relacionada con la satisfacción de los pacientes, se tuvo en cuenta el indicador de rendimiento de los centros de salud, usado por la Consejería de Sanidad, denominado «demora». Este indicador pretende evaluar el tiempo de espera de los pacientes para conseguir una cita. Concretamente, mide el porcentaje de agendas de los médicos que tienen disponibilidad, para las citas de los pacientes, en un plazo menor o igual a una jornada. La conveniencia de incluir esta variable se basa tanto en su carácter objetivo como en que la investigación ha mostrado una relación significativa y negativa entre el tiempo de espera por parte de los pacientes y su sastisfacción^21^.

Los datos referentes al compromiso organizativo afectivo se obtuvieron a través de cuestionarios autoadministrados, repartidos por los investigadores, en mayo de 2019. Los cuestionarios iban dirigidos a todos los médicos y enfermeros de los 40 centros de atención primaria del Área de Salud de la isla Gran Canaria. Posteriormente, entre los meses de junio a septiembre, los cuestionarios fueron recogidos conforme iban siendo completados. Para la medición del compromiso organizativo afectivo se utilizó la escala del cuestionario de Meyer y Allen^17^, compuesta por seis ítems. Se contestaba mediante una escala tipo Likert de siete puntos en la que 1 era muy en desacuerdo y 7 muy de acuerdo.

A partir de fuentes secundarias, se incluyeron datos sobre las siguientes variables:

• Entorno socioeconómico de la población adscrita a los centros de atención primaria: hace referencia a los aspectos demográficos y socioeconómicos de los pacientes. Incluye las siguientes variables para cada centro de salud: población total, peso de la población mayor de 65 años y renta por hogar. Los datos se obtuvieron a través de la información ofrecida por el Instituto Nacional de Estadística, excepto la población total de usuarios de cada centro de salud que fue proporcionada por el personal técnico de la Consejería de Sanidad.
• Características de los centros de salud: hace referencia a la ratio de pacientes por profesional, la ratio de pacientes mayores de 65 años por profesional y el presupuesto por paciente. Las variables fueron calculadas a partir de los datos cedidos por el personal técnico de la Consejería de Sanidad. Para su análisis se calculó su logaritmo.

Además, tras finalizar el análisis cuantitativo, se realizaron entrevistas estructuradas a cinco directores de centros de salud para profundizar en los resultados encontrados.

### Tamaño muestral

Mediante un muestreo no probabilístico por conveniencia, se recibieron 617 respuestas de una población de 1.134 profesionales sanitarios de centros de atención primaria. Tras descartarse 12 cuestionarios, por contener fallos u omisiones, la muestra definitiva fue de 605 profesionales (29,26% hombres y 66,90% mujeres); 46% eran médicos y 54% enfermeros. De todos los centros se obtuvo como mínimo una participación del 50% de sus profesionales. La antigüedad media en el centro de salud fue 10,5 años y en la organización 20,8 años. En cuanto a los usuarios de los centros de salud, la muestra fue de 1.537 pacientes (35,39% hombres y 64,60% mujeres) de todos los centros de salud. El muestreo realizado por la Consejería de Sanidad de la Administración Autonómica del Gobierno de Canarias también fue un muestreo por conveniencia.

### Declaración ética

Los individuos que han participado en el estudio lo hicieron de forma voluntaria. Se garantizó la confidencialidad de los participantes del estudio, no revelando ningún dato que favorezca la identificación de los mismos.

Se cumplen con los principios éticos establecidos en la Declaración de Helsinki y con la Ley Orgánica 15/1999, de 13 de diciembre, de Protección de Datos de Carácter Personal.

### Análisis de datos

No se incluyeron datos ausentes. La fiabilidad para escala que medía el compromiso organizativo afectivo se calculó utilizando el alfa de Cronbach (valor mínimo de 0,70), la fiabilidad compuesta (valor mínimo de 0,70) y la varianza extraída media (valor mínimo de 0,50)^22^. La dimensionalidad y la validez convergente de la escala se analizó mediante un análisis factorial con rotación varimax y método de máxima verosimilitud.

Con el objetivo de averiguar cuáles son las variables que influyen en la satisfacción de los pacientes y en el indicador de demora, se utilizó el modelo lineal de regresión múltiple. Esta técnica permite analizar la influencia de un conjunto de variables sobre una variable dependiente. Como variable independiente se incluyó el compromiso organizativo afectivo. Así mismo, se incluyeron como variables de control las referidas al entorno socioeconómico de los centros de salud (población total, peso de la población mayor de 65 años y renta por hogar) y las concernientes a las características de los centros de salud (presupuesto por paciente, ratio de paciente por profesional, ratio de pacientes mayores de 65 años por profesional).

**Figura 1:**
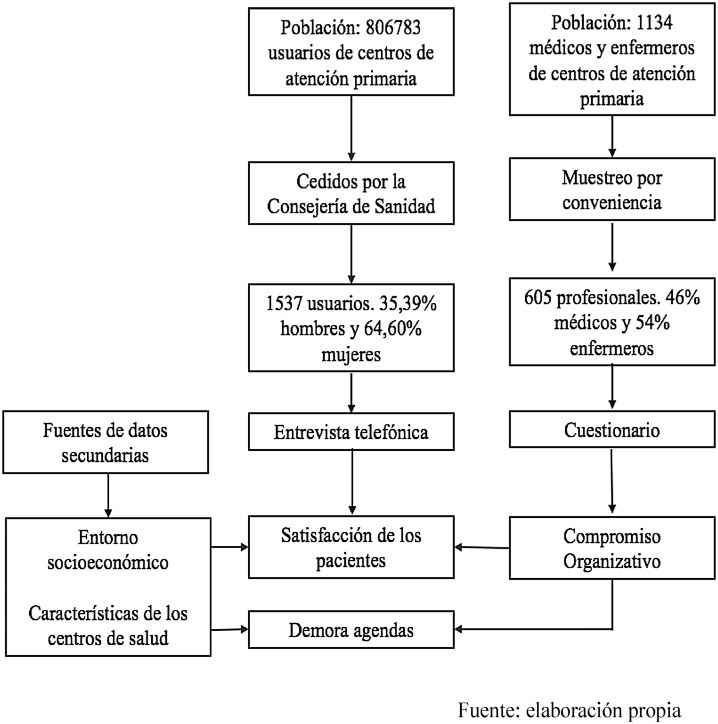
Esquema general del estudio Fuente: elaboración propia.

La figura 1 representa el esquema del estudio, desde la recogida de datos hasta su análisis. A partir de los datos de encuesta y de fuentes secundarias, se pretende conocer si el compromiso organizativo del personal sanitario influye en la satisfacción de los usuarios de atención primaria.

## Resultados

La variable independiente, el compromiso organizativo afectivo, mostró las siguientes estadísticas: media (5,15); desviación típica (1,33), mínimo (1); y máximo (7). Su distribución por cuartiles fue: primer cuartil (5,02), segundo cuartil (5,24) y tercer cuartil (5,47). La fiabilidad de la escala, a partir del alfa de Cronbach, fue 0,87. La fiabilidad compuesta y la varianza extraída media fueron 0,88 y 0,55, respectivamente. En cuanto a la dimensionalidad, el análisis factorial generó un único factor y constató que todos los ítems mostraban cargas factoriales superiores a 0,50. Los resultados se recogen en la tabla 1.

**Tabla 1:** Resultados del análisis factorial

Compromiso organizativo afectivo	Carga factorial
Compromiso organizativo afectivo 1	0,65
Compromiso organizativo afectivo 2	0,63
Compromiso organizativo afectivo 3	0,78
Compromiso organizativo afectivo 4	0,75
Compromiso organizativo afectivo 5	0,79
Compromiso organizativo afectivo 6	0,85
Porcentaje de varianza explicada	62,47
Valor propio	3,75
Índice KMO	0,89
Test de esfericidad de Bartlett	1.722,21
Significación	0,00

En la tabla 2 podemos observar los datos de las variables dependientes y de control.

**Tabla 2:** Datos de las variables de control y de la variable dependiente

*Variables de control del entorno de los centros de salud*	Media (dt)	Mediana	Mínimo	Máximo
Renta media por hogar (euros)	26.218,76 (4.782,44)	25.084,33	21.526,13	42.044,13
Pacientes totales	20.686,74 (14.706,34)	20.762	1.464	75.399
Población > 65 años	3.360 (2.073)	3.154	334	8.586
Peso población > 65 años	0,17 (0,03)	0,18	0,11	0,25
*Variables de control del centro de salud*				
Pacientes por profesional	649,79 (138,77)	664,93	183,00	839,78
Presupuesto por paciente (euros)	443,63 (154,34)	407,39	198,04	977,69
Población > 65 años por profesional	110,90 (25,97)	108,52	41,75	175,81

*Variables dependientes*				
Satisfacción de los pacientes	8,26 (0,59)	8,22	5,80	10,00
Demora (%)	16,86 (17,40)	14,29	0,00	100,00

dt: desviación típica.

Con base en lo anterior, se aplicó el modelo lineal de regresión múltiple tomando como variable dependiente la satisfacción del paciente. Se realizó un primer modelo donde se incluyeron las variables de control referidas al entorno socioeconómico de la población asignada a los centros de salud: población total, peso de la población mayor de 65 años y renta por hogar. El modelo no resultó estadísticamente significativo, indicando que ninguna de ellas contribuía a explicar la satisfacción del paciente.

Teniendo en cuenta lo anterior, se procedió a realizar un segundo modelo donde se incluyeron como variables de control aquellas relacionadas con las características de los centros de salud: presupuesto por paciente, ratio pacientes por profesional y ratio pacientes mayores de 65 años por profesional. En este modelo (tabla 3), resultaron significativas las variables presupuesto por paciente (β: 0,43; p: 0,05) y la ratio paciente por profesional (β: 0,78; p: 0,00). La R^2^ ajustada es de 0,15, lo que indica que la satisfacción del paciente se ve explicada en un 15%.

**Tabla 3:** Regresión lineal múltiple para satisfacción del paciente

Satisfacción del paciente	Modelo 2		Modelo 3	
Variables	Coeficientes estandarizados	Sig.	Coeficientes estandarizados	Sig.
Presupuesto por paciente	0,43	0,05	0,34	0,09
Ratio pacientes por profesional	0,78	0,00	0,61	0,02
Ratio pacientes > 65 años por profesional	-0,26	0,19	-0,19	0,31
Compromiso afectivo			0,39	0,01
R^2^	0,22	0,03	0,36	0,04
R^2^ ajustada	0,15		0,28	
F	3,24		4,72	

Posteriormente se realizó un tercer modelo donde se incluyó, además, como variable independiente el compromiso organizativo afectivo. En el tercer modelo (tabla 3), la R^2^ ajustada aumentó a 0,28, resultando significativas las variables ratio pacientes por profesional (β: 0,61; p: 0,02) y compromiso organizativo afectivo (β: 0,39; p: 0,01).

A continuación, se aplicó el modelo lineal de regresión múltiple, empleando como variable dependiente el indicador de demora en las agendas de los profesionales. Se realizó el primer modelo incluyendo nuevamente las variables relacionadas con el entorno socioeconómico de los centros de salud. El modelo no resultó estadísticamente significativo. Se procedió a realizar el segundo modelo (tabla 4) con las variables relacionadas con las características de los centros de salud. Resultó significativa, en sentido negativo, la ratio de pacientes por profesional (β: -0,66; p: 0,01), obteniéndose una R^2^ ajustada de 0,15. A continuación se realizó el tercer modelo añadiendo el compromiso organizativo afectivo. En este modelo (tabla 4) fueron significativas, ambas con influencia negativa, la ratio de pacientes por profesional (β: -0,50; p: 0,05) y la variable compromiso afectivo (β: -0,36; p: 0,02). La R^2^ ajustada fue de 0,26.

**Tabla 4:** Regresión lineal múltiple para demora

Demora	Modelo 2		Modelo 3	
Variables	Coeficientes estandarizados	Sig.	Coeficientes estandarizados	Sig.
Presupuesto por paciente	-0,25	0,25	-0,16	0,43
Ratio pacientes por profesional	-0,66	0,01	-0,50	0,05
Ratio pacientes > 65 años por profesional	0,10	0,62	0,03	0,87
Compromiso afectivo			-0,36	0,02
R^2^	0,21	0,04	0,34	0,01
R^2^ ajustada	0,15		0,26	
F	3,16		4,31	

Debido a que la contribución del compromiso organizativo afectivo al indicador de demora resultó en sentido contrario al esperado (más compromiso afectivo implica menos agendas con disponibilidad para citas en un tiempo igual o inferior a una jornada), se entrevistó a cinco directores de centros de salud para discutir este resultado. Todos coincidieron en que la explicación era que los profesionales más comprometidos dedicaban más tiempo a sus pacientes y, por ello, tenían menos disponibilidad para citas en el plazo recogido en el indicador. Comentaron que cuando las consultas de los médicos llevan retraso, y no tienen disponibilidad a corto plazo, es porque los profesionales dedican más tiempo a los pacientes y que estos se sienten satisfechos, ya que creen que se les dedica más tiempo y atención. En este sentido, la correlación entre la satisfacción de los pacientes y el porcentaje de agendas con disponibilidad para citas en un tiempo igual o inferior a una jornada fue negativa (r: –0,62, p: 0,00). De hecho, los directores resaltaron que los usuarios pueden cambiarse de médico si quieren (escogiendo a aquellos con más disponibilidad para citas en su agenda), pero no lo hacen, ya que dan más valor a la atención recibida que a que se les reciba de un día para otro. Así, los médicos que dedican más tiempo a los pacientes son los preferidos en los centros de salud.

## Discusión

El análisis de los datos muestra la influencia positiva que tiene el compromiso organizativo afectivo sobre la satisfacción de los pacientes (β: 0,39; p: 0,01), lo que significa que cuanto mayor es el apego emocional y la implicación de los profesionales sanitarios con su centro de salud, mayor es la satisfacción de los pacientes. Este resultado coincide con los de otros estudios de sectores diferentes al sanitario^14^, ^15^, ^16^, que muestran la influencia positiva que ejerce el compromiso, en particular la dimensión afectiva, del empleado sobre la satisfacción del cliente.

El compromiso afectivo influye de manera negativa en el indicador demora en las agendas (β: -0,36; p: 0,02). Este resultado parece ir en contra del anterior hallazgo, y de lo que recoge la literatura, referente a que el tiempo de espera por parte de los pacientes está relacionado de manera negativa con su satisfacción^21^. Las entrevistas realizadas clarifican el resultado, ya que, según los directores, contar con mucha disponibilidad para citas en tan corto plazo (una jornada o menos) refleja que el tiempo que se dedica a los pacientes es insuficiente para estos. Así, contar con un alto compromiso implicaría dedicar más tiempo a los pacientes y, consecuentemente, no tener la disponibilidad que mide el indicador. La correlación negativa entre este y la satisfacción de los pacientes (r: –0,62, p: 0,00) es consistente con esta explicación.

El anterior resultado pone de manifiesto lo importante que es usar indicadores de evaluación adecuados. En este caso, el indicador demora es usado para la evaluación de los centros de salud. Nuestros resultados confirman que va en sentido contrario a la satisfacción de los pacientes y que son, precisamente, los profesionales con menos compromiso los que mejor se posicionan en el indicador. En palabras de unos de los entrevistados, este indicador parece reflejar más la rapidez con la que los profesionales despachan a los pacientes.

La ratio pacientes por profesional resultó significativa para la satisfacción de los pacientes (β: 0,61; p:0,02). El sentido positivo de la relación también parece contrario a lo que podría esperarse. La explicación podría residir en lo que ya hemos comentado referente a que, más que el cupo de pacientes que tienen los profesionales, lo que realmente hace que los usuarios estén satisfechos es el tiempo que se les dedica. Y, según las entrevistas, los que más lo hacen son los más preferidos y los que tienen más pacientes.

Si bien el presupuesto por paciente de los centros de salud parece influir en la satisfacción de los pacientes (β: 0,43; p: 0,05 de la regresión sin la variable compromiso), este pierde relevancia (p > 0,05), cuando se incluye el compromiso afectivo de los profesionales. Hay que tener en cuenta que la mayor parte del presupuesto de los centros lo componen sus profesionales. Este resultado, y la influencia positiva encontrada de la ratio de pacientes por profesional, reflejan que lo realmente importante para la satisfacción de los pacientes es el compromiso de los profesionales más que la cantidad de estos.

Las variables del entorno socioeconómico de la población (población total asignada, peso de población mayor de 65 años, y renta por hogar) no resultaron significativas, a pesar de que la literatura refleja que el nivel socioeconómico de la población ejerce efecto sobre la actividad del sistema sanitario y sus profesionales^23^, ^24^. Así, en los centros de atención primaria la satisfacción de los pacientes depende más de los recursos humanos de los propios centros, concretamente del compromiso afectivo de su personal y del tiempo que dedican a los pacientes, que de la cantidad y el nivel socioeconómico de los usuarios adscritos a los centros.

Tanto en el sector privado como en el público, los recursos humanos y económicos para la prestación de cualquier servicio siempre son finitos. De hecho, la sanidad pública siempre demanda más recursos. Por ello, mejorar la satisfacción de los pacientes a base de más recursos tiene un límite. Esta investigación muestra que la misma se logra, además, con una plantilla de profesionales sanitarios comprometidos. Los gestores sanitarios tienen a su disposición las prácticas de recursos humanos que han mostrado influir en el compromiso de los trabajadores. A su vez, la medición periódica del compromiso de estos es, con la ayuda de la tecnología actual, una acción sencilla y cuyos resultados pueden ayudar a implantar acciones que finalmente redundan en la mejora de la satisfacción de los pacientes.

Una de las limitaciones del estudio es la tasa de respuesta de los profesionales sanitarios, que, aunque técnicamente es aceptable^25^, presenta limitaciones a la hora de generalizar los resultados. A su vez, la muestra de pacientes también es una de conveniencia. Otra de las limitaciones es que el diseño de este estudio no muestra una relación de causalidad entre las variables utilizadas y la satisfacción del paciente, sino la asociación entre las variables estudiadas.

Finalmente, como futuras líneas de investigación se plantea evaluar el papel del compromiso organizativo de los profesionales sanitarios en otros indicadores del rendimiento de los centros de salud, como puede ser el cumplimiento de objetivos relacionados con la ejecución de programas sanitarios. A su vez, estudiar el rol que pueden desempeñar los medios tecnológicos actuales, en la relación de los profesionales con los pacientes, es otro aspecto a considerar en la búsqueda de la satisfacción de estos últimos.

## Conclusión

La satisfacción de los pacientes de los centros de salud está relacionada con el compromiso afectivo de los profesionales sanitarios. Aunque el diseño de este estudio no muestra una relación causal, la literatura sí constata que los trabajadores comprometidos tienen un mayor rendimiento, por lo que, de cara a garantizar la satisfacción de los pacientes, los gestores sanitarios deberían esforzarse por contar con una plantilla comprometida.

**Lo conocido sobre el tema**

En el sector sanitario público, la satisfacción de los pacientes es considerada un indicador de la calidad de los servicios. En otros entornos, la satisfacción del cliente se relaciona directamente con el compromiso organizativo de los trabajadores, hecho que no ha sido estudiado en el ámbito sanitario. Por tanto, en este estudio se pretende conocer si en el ámbito sanitario el compromiso organizativo de los trabajadores influye en la satisfacción de los usuarios y, en definitiva, en la calidad del servicio.

**Qué aporta este estudio**

En este estudio ha quedado evidenciada la influencia que ejerce el compromiso organizativo de los profesionales sanitarios de atención primaria en la satisfacción de los pacientes. Así como se muestra la influencia que ejerce el compromiso organizativo sobre la demora en las agendas de los profesionales. Por otro lado, ha quedado evidenciado que el entorno socioeconómico de los centros de salud no influye en la satisfacción de los pacientes.

## Financiación

Este trabajo no ha recibido ningún tipo de financiación.

## Conflicto de intereses

Los autores declaran no tener ningún conflicto de intereses.
